# Targeting hepatic CREBZF protects against atherosclerosis via exosomal miR-210-3p mediated inhibition of endothelial inflammation

**DOI:** 10.1016/j.gendis.2026.102212

**Published:** 2026-04-22

**Authors:** Jinxuan Wang, Xiaoqi Zhou, Yuanyuan Liu, Jianxiong Xu, Yanzhu Long, Shuai Hu, Cheng Wang, Liqiong Yan, Chen Yang, Langtao Wang, Liping Luo, Tao Yu, Yang Shen, Yu Li, Xiangyu Zhou, Guixue Wang, Xiaozhen Dai

**Affiliations:** a Aging Mechanisms and Interventions Key Laboratory of Sichuan Province, School of Basic Medical Sciences, Chengdu Medical College, Chengdu, Sichuan 610500, China; b School of Laboratory Medicine, Chengdu Medical College, Chengdu, Sichuan 610500, China; c College of Health Management, Xihua University, Chengdu, Sichuan 610039, China; d Key Laboratory of Target Discovery and Protein Drug Development in Major Diseases of Sichuan Higher Education Institutes, School of Biological Sciences and Technology, Chengdu Medical College, Chengdu, Sichuan 610500, China; e School of Clinical Medicine, Chengdu Medical College, Chengdu, Sichuan 610500, China; f Institute of Biomedical Engineering, West China School of Basic Medical Sciences & Forensic Medicine, Sichuan University, Chengdu, Sichuan 610041, China; g CAS Key Laboratory of Nutrition, Metabolism and Food Safety, Shanghai Institute of Nutrition and Health, University of Chinese Academy of Sciences, Chinese Academy of Sciences, Shanghai 200031, China; h Department of Cardiology, The Affiliated Hospital of Southwest Medical University and Key Laboratory of Medical Electrophysiology, Ministry of Education, Institute of Cardiovascular Research and Institute of Metabolic Diseases, Southwest Medical University, Luzhou, Sichuan 646000, China; i Key Laboratory for Biorheological Science and Technology of the Ministry of Education, National Local Joint Engineering Lab for Vascular Implants, College of Bioengineering, Chongqing University, Chongqing 400044, China; j Hubei Shizhen Laboratory, Hubei University of Chinese Medicine, Wuhan, Hubei 430065, China

**Keywords:** Atherosclerosis, CREBZF, Exosomes, Liver-vascular crosstalk, miRNA

## Abstract

Cardiovascular diseases (CVDs) continue to be the primary cause of mortality globally. Abnormal liver function has been widely recognized as an independent risk factor for atherosclerotic CVDs. However, how alterations in liver function affect distal vascular function remains largely unknown. CREBZF, a bZIP transcription factor of the CREB/ATF family, has been identified as a driver of hepatic steatosis and fibrosis. Here, we provide the first demonstration that hepatic CREBZF acts as a critical regulator driving atherosclerotic progression. *In vivo*, hepatic-specific deletion of CREBZF attenuates endothelial inflammation and reduces macrophage infiltration, thereby ameliorating plaque formation in both high-fat diet-fed and spontaneous atherosclerotic mouse models. *In vitro*, overexpression or knockdown of CREBZF in hepatocytes correspondingly promotes or attenuates endothelial inflammation. Mechanistically, hepatocyte CREBZF regulates endothelial inflammation via exosomes, an effect independent of cholesterol metabolism or of soluble cytokines. Furthermore, CREBZF dynamically modulates the expression of exosomal miR-210-3p and its subsequent levels in recipient endothelial cells. miR-210-3p, in turn, suppresses endothelial inflammation by targeting C-Rel, a component of the NF-κB transcription factor family. Importantly, the pro-inflammatory effect of exosomes derived from CREBZF-overexpressing hepatocytes on endothelial cells is abrogated by a miR-210-3p mimic. Collectively, these findings unveil a novel mechanism by which hepatic CREBZF regulates atherosclerosis through liver-vascular crosstalk mediated by the exosomal miR-210-3p/C-Rel signaling axis, suggesting a potential therapeutic strategy for atherosclerotic CVDs.

## Introduction

Cardiovascular diseases (CVDs) continue to be the primary cause of mortality globally.^1^, ^2^, ^3^ Atherosclerosis (AS), the pathological basis of CVDs, underlies most major cardiovascular events. The core mechanism of atherosclerosis involves a vicious cycle of “injury-inflammation-lipid deposition”. Cardiovascular risk factors, such as hyperlipidaemia and hypertension, induce endothelial injury. This, in turn, triggers an inflammatory response that disrupts the endothelial barrier, thereby promoting lipid deposition within the vascular wall and ultimately initiating plaque formation.^4^, ^5^, ^6^, ^7^ The plaque rupture and subsequent acute thrombosis are the primary causes of death and disability in affected patients.

Accumulating evidence suggests that liver dysfunction and endothelial impairment are associated with multiple atherosclerosis markers, independent of traditional risk factors.^8^, ^9^, ^10^, ^11^ In nonalcoholic fatty liver disease (NAFLD), abnormal intrahepatic triglyceride accumulation disrupts systemic lipid homeostasis, promotes inflammation, and elevates oxidative stress. Consequently, NAFLD has emerged as an independent risk factor for atherosclerotic CVDs.^12^^,^^13^ Beyond its metabolic roles, the liver contributes to atherosclerosis pathogenesis through the release of extracellular vesicles (EVs) and soluble cytokines. These mediators systemically regulate lipid metabolism, inflammatory responses, vascular function, and intercellular communication.^14^, ^15^, ^16^ Currently, statin drugs have been primarily used to target the liver for lipid lowering and atherosclerosis improvement. Although lipid levels in most patients can be reduced to target ranges, some individuals still experience recurrent cardiovascular events.^17^^,^^18^ Thus, further clarification of the critical genes and mechanisms controlling liver-vascular crosstalk is necessary to identify more effective therapeutic targets.

CREBZF is a transcription factor belonging to the CREB family. It forms homo- or heterodimers with itself or other CREB family members through its basic leucine zipper (bZIP) domain and typically functions as a transcriptional repressor.^19^ In hepatocytes, the transcriptional activity of CREBZF is regulated by insulin and contributes to the development of NAFLD and the induction of liver fibrosis. Multiple studies have demonstrated that liver-specific knockout of CREBZF alleviates hepatocyte inflammation and reduces lipid accumulation.^19^, ^20^, ^21^ Furthermore, Liu et al demonstrated that overnutrition-induced CREBZF links the proinflammatory activation of adipose tissue macrophage to insulin resistance.^22^ While CREBZF regulates hepatic inflammation and lipid metabolism, whether it concurrently impacts distal vascular function remains unknown. The mechanisms by which CREBZF mediates communication between hepatocytes and vascular endothelial cells (ECs) remain unclear.

Here, we propose a novel perspective on the regulatory mechanism of organ crosstalk in the pathogenesis of hepatocyte inflammation and atherosclerosis. Functional manipulation experiments have established the pivotal role of hepatocyte CREBZF in regulating endothelial inflammation and atherogenesis. *In vivo* and *in vitro* studies have demonstrated that (1) hepatic-specific CREBZF deficiency ameliorates plaque formation by attenuating endothelial inflammation; (2) hepatic CREBZF regulates endothelial inflammation through hepatocyte-derived exosomal miR-210-3p; and (3) identified miR-210-3p as a newly discovered protective mediator in ECs, mitigating the proinflammatory effects induced by hepatocyte CREBZF.

## Materials and methods

### Animal experiments

All experimental procedures involving animals received prior approval from the Ethics Committee of Chengdu Medical College. Mice were raised in specific pathogen-free (SPF) facilities, with the room temperature maintained at 24–25 °C and relative humidity at approximately 55%. To generate liver-specific CREBZF knockout mice on an *ApoE*^−/−^ background, homozygous *CREBZF*-floxed mice were crossed with Alb-Cre transgenic mice. Both strains were kindly provided by the laboratory of Professor Yu Li (Chinese Academy of Sciences). The resulting *CREBZF* LKO mice and their control littermates (*CREBZF*^fl/fl^) were crossed with *ApoE*^−/−^ mice to generate *CREBZF* LKO; *ApoE*^−/−^ and *CREBZF*^fl/fl^; *ApoE*^−/−^ mice. Genotyping was performed on DNA extracted from the tails of three-week-old mice. For genotyping, the primer sequences for the *CREBZF*-Flox allele were as follows: 5′-GCTTGCAGTTTAGAGAGAAACAGC-3′; 5′-CAGCCAGAGTATCGCGAGATTC-3′. Band sizes: *CREBZF*^fl/fl^, 318 bp; Wild type, 248 bp. The primer sequences for Alb-Cre were as follows: 5′-TTGGCCCCTTACCATAACTG-3’; 5′-GAAGCAGAAGCTTAGGAAGATGG-3′. Band size: Alb-Cre, 400 bp.

All experimental mice were male. To investigate the role of hepatic CREBZF in atherosclerosis, 8-week-old *CREBZF* LKO; *ApoE*^−/−^ and control mice were fed either a normal chow diet (ND) or a high-fat diet (HFD) (D12108C, Changzhou Shuyi Shuer Biotechnology) for 12 weeks according to previously described methods.^7^ In a separate long-term cohort, 8-week-old *CREBZF* LKO; *ApoE*^−/−^ and control mice were maintained under routine conditions for 12 months. Following the dietary interventions, the mice were anaesthetized, blood samples were collected, and systemic perfusion with phosphate buffer saline (PBS) was performed via the heart. Heart and aortic tissues were then collected for staining and analysis.

### Cell culture and treatments

Hepatocytes were cultured in DMEM (Gibco, Cat. # 11995065), while human umbilical vein endothelial cells (HUVECs) were grown in RPMI 1640 medium (Gibco, Cat. # 11875093). Unless otherwise specified for specific experimental requirements, cells were routinely cultured in complete medium containing 10% fetal bovine serum (FBS) and 1% penicillin-streptomycin. AML12 cells were transfected with siRNA using Lipofectamine™ RNAiMAX transfection reagent (Thermo Fisher, Cat. # 13778150) as previously described.^23^

### Purification and profiling of exosomes derived from hepatocytes

Prior to medium preparation, FBS was ultracentrifuged (120,000×*g*, 14 h, 4 °C) to generate exosome-depleted serum. HepG2 cells were then treated with adenovirus (Ad-CREBZF) or control adenovirus (Ad-NC) for 48 h, followed by replacement with medium containing exosome-depleted serum and further cultured for 24 h. Exosomes were isolated from conditioned medium by differential centrifugation. Cellular components were removed through sequential centrifugation at 300×*g*, 2000×*g*, and 10,000×*g* at 4 °C. The filtered (0.22 μm) supernatant was subjected to centrifugation at 120,000×*g* for 70 min. After resuspension, the pellet was subjected to the aforementioned procedure again to yield an exosome-enriched fraction. The diameter and morphology of the exosomes were subsequently characterized using nanoparticle tracking analysis (NTA) and transmission electron microscopy (TEM) (both from Malvern Panalytical, UK), respectively.

### Conditioned medium-based hepatocyte-EC interaction assay

HepG2 cells were treated with either CREBZF-overexpressing adenovirus (Ad-CREBZF) or a control adenovirus (Ad-NC) for 48 h. The culture medium was then discarded and replaced with DMEM supplemented with exosome-depleted serum and incubated for 24 h. The conditioned medium was collected and used to treat HUVECs. After treatment, HUVECs were harvested for mRNA or protein analysis.

### Exosome uptake assay

PKH67-labeled hepatocyte-derived exosomes were incubated with HUVECs in complete growth medium at 37 °C for 12 h. Nuclear counterstaining was performed with Hoechst 33342 (10 min, 25 °C, in the dark). Exosome uptake was assessed by confocal laser scanning microscopy.

### Immunofluorescence

After permeabilization (0.25% Triton X-100, 10 min), cardiac sections were subjected to blocking with 5% BSA (BIOFROXX, Cat. # 4240GR250) in PBS (1 h, 25 °C). Primary antibody incubation was performed overnight using anti-MCP1 and anti-ICAM1 (ProteinTech, Cat. # 66272-1-Ig and # 82827-8-RR) diluted in PBS with 2.5% BSA at 4 °C. Following PBS washes, the sections were incubated with fluorescent secondary antibodies (ProteinTech, Cat. # SA00013-2 and # SA00009-1) for 1 h at 25 °C, and nuclei were counterstained with DAPI (Cell Signaling Technology, Cat. # 4083S). Sections were finally washed, mounted, and imaged.

### Western blot analysis

After centrifugation, the clarified supernatant of cell and tissue lysates was collected. Proteins were separated by SDS-PAGE using a 5% stacking gel and a 10%–15% resolving gel. After separation, blotting was performed at a constant current of 250 mA at 4 °C for 90 min to transfer proteins onto a PVDF membrane (Millipore, Billerica, MA). The primary antibodies used were as follows: p65, phospho-p65 and ICAM1 (Cell Signaling Technology, Cat. # 6956, Cat. # 3033, Cat. # 4915), VCAM1 (Beyotime, Cat. # AF1021), TNF-α (ABclonal, Cat. # A11534), CREBZF (Abcam, Cat. # AB28700), GAPDH (Zen Bioscience, Cat. # 200306-7E4), β-actin, CD81, and CD63 (Proteintech, Cat. # 66009-1, Cat. # 27855-1, Cat. # 25682-1), and TSG101 (Abways, Cat. # CY5985). Following three TBST washes, the samples were then incubated with an appropriate dilution of HRP-conjugated secondary antibody: anti-mouse IgG-HRP or anti-rabbit IgG-HRP (Proteintech, Cat. # RGAM001, # SA00001-2). Chemiluminescent signals from protein bands were captured with a commercial substrate (BioSharp, Cat. # BL520B).

### RNA isolation and quantification

The cells were lysed with TRIzol Universal Reagent (TIANGEN, Cat. # DP424). The lysate was transferred to RNase-free microcentrifuge tubes. Chloroform was added to the lysate, which was vortexed vigorously, and then centrifuged at 4 °C. The aqueous phase was aspirated, isopropanol was added, the precipitated RNA was washed twice with 75% ethanol and dried. The RNA in 50 μL of RNase-free water was dissolved. Total RNA from the exosomes was extracted via a miRNeasy Micro Kit (QIAGEN, Cat. # 1071023). Exosomal miRNA levels were normalized against cel-miR-39 (an external control), and 0.5 pmol per sample was added to 100 μg of exosomes in advance. cDNA was synthesized from mRNA and miRNA using the Evo M-MLV RT Mix Kit and the miRNA 1st Strand cDNA Synthesis Kit (both from Accurate Biotechnology), respectively. The endogenous U6 RNA or spike-in cel-miR-39-3p RNA was used to normalize the cellular or exosomal miRNA. The primers used in q-PCR assays are shown in Table S1.

### Transfection of the miRNA mimic or inhibitor

miRNA mimics or inhibitors (GenePharma, Shanghai, China) were transfected into cells at 20 nM using Lipofectamine™ RNAiMAX. The miRNA and Lipofectamine™ RNAiMAX transfection reagent were diluted separately with Opti-MEM™ (Thermo Fisher Scientific, USA), and then the miRNA-containing mixture was added to the Lipofectamine™ RNAiMAX mixture. The mixture was incubated for 5–10 min to allow complex formation. The complexes were added to HUVECs seeded in a 12-well plate, cultured for 24 h, and then replaced with fresh medium. Culturing was continued for another 24 h before harvesting for analysis.

### Measurement of hepatic cholesterol and triglyceride levels

We determined the concentrations of total cholesterol (TC) and triglycerides (TG) in mouse liver tissue using commercial kits per the provided protocols (both from Applygen, Cat. # E1015-105, Cat. # E1013-105).

### ELISA

Following collection and clotting (30 min, 25 °C), mouse blood was centrifuged (2000×*g*, 15 min, 4 °C) to isolate serum. Determination of serum IL-6, TNF-α, IL-1β, and MCP1 concentrations was performed with specific ELISA kits (RUIXIN BIOTECH: IL-6, Cat. # RXW203049M; TNF-α, Cat. # RXW202412M; IL-1β, Cat. # RXW203063M; MCP1, Cat. # RXW202991M) according to the kit instructions. The assay procedure involved incubating samples and standards in coated plates, washing, sequential addition of biotinylated detection antibody and streptavidin-HRP, TMB-based color development, and acid stop. Absorbance at 450 nm was measured, and concentrations were calculated against a standard curve.

### Plasma parameters and lipoprotein profiles

After an overnight fast, blood samples were obtained from mice by retro-orbital bleeding for serum preparation. We determined the concentrations of TC, TG, low-density lipoprotein (LDL), and high-density lipoprotein (HDL) using the BS-460 Veterinary Biochemical Analyzer (Mindray, Shenzhen, China).

### Statistical analysis

Data presentation and analysis were performed using GraphPad Prism (version 8.0.2; GraphPad Software, San Diego, CA, USA). The statistical results for all comparisons between two groups in this study were analyzed using an unpaired two-tailed Student's *t*-test. Data are presented as mean ± SEM. ns, not significant; ∗*p* < 0.05, ∗∗*p* < 0.01, ∗∗∗*p* < 0.001.

## Results

### Hepatic CREBZF deficiency attenuates atherosclerotic development in *ApoE*^−/−^ mice

CREBZF has been demonstrated to accelerate hepatic lipid droplet formation and exert proinflammatory effects in both diet-induced insulin-resistant and genetically obese mouse models.^20^^,^^22^ Having established the role of CREBZF in hepatocytes, we next investigated whether it influences vascular cells through inter-organ communication. To determine whether hepatic CREBZF is involved in AS, we first confirmed that ApoE deficiency, the basis of a classic AS mouse model, did not alter its basal expression. However, upon inducing AS in *ApoE*^−/−^ mice fed with a 12-week high-fat diet (HFD), hepatic CREBZF expression was markedly elevated compared to that in chow-fed controls (Fig. S2). This finding implicates hepatic CREBZF as a potential contributor to AS progression.

To create mice with a hepatocyte-specific knockout of CREBZF (*CREBZF* LKO), we crossed CREBZF-floxed (*CREBZF*^fl/fl^) mice with Alb-Cre transgenic mice (Fig. S1A). To generate *CREBZF* LKO; *ApoE*^−/−^ mice for atherogenesis studies, *CREBZF* LKO mice were crossed with *ApoE*^−/−^ mice. Quantitative analysis of livers isolated from *CREBZF* LKO mice revealed an 84% knockout efficiency of CREBZF (Fig. 1A). We observed the formation of plaques in *CREBZF* LKO; *ApoE*^−/−^ mice under both HFD-induced and spontaneous conditions (Fig. S1B and S1C). After 12 weeks of HFD feeding, the total atherosclerotic area in *CREBZF* LKO; *ApoE*^−/−^ mice was significantly reduced compared with that in *CREBZF*^fl/fl^; *ApoE*^−/−^ mice, as shown by Oil Red O (ORO) staining analysis of the aortas (Fig. 1B and C). Aortic root staining also revealed that the plaque area was significantly attenuated in *CREBZF* LKO; *ApoE*^−/−^ mice (Fig. 1D and E). We concurrently assessed spontaneous plaque development and found that after 12 months on a normal diet (ND), the lesion area in both the aorta and the aortic root was strikingly reduced in *CREBZF* LKO; *ApoE*^−/−^ mice (Fig. 1F–I), further suggesting that targeted intervention against hepatic CREBZF can ameliorate atherogenesis.

**Figure 1:**
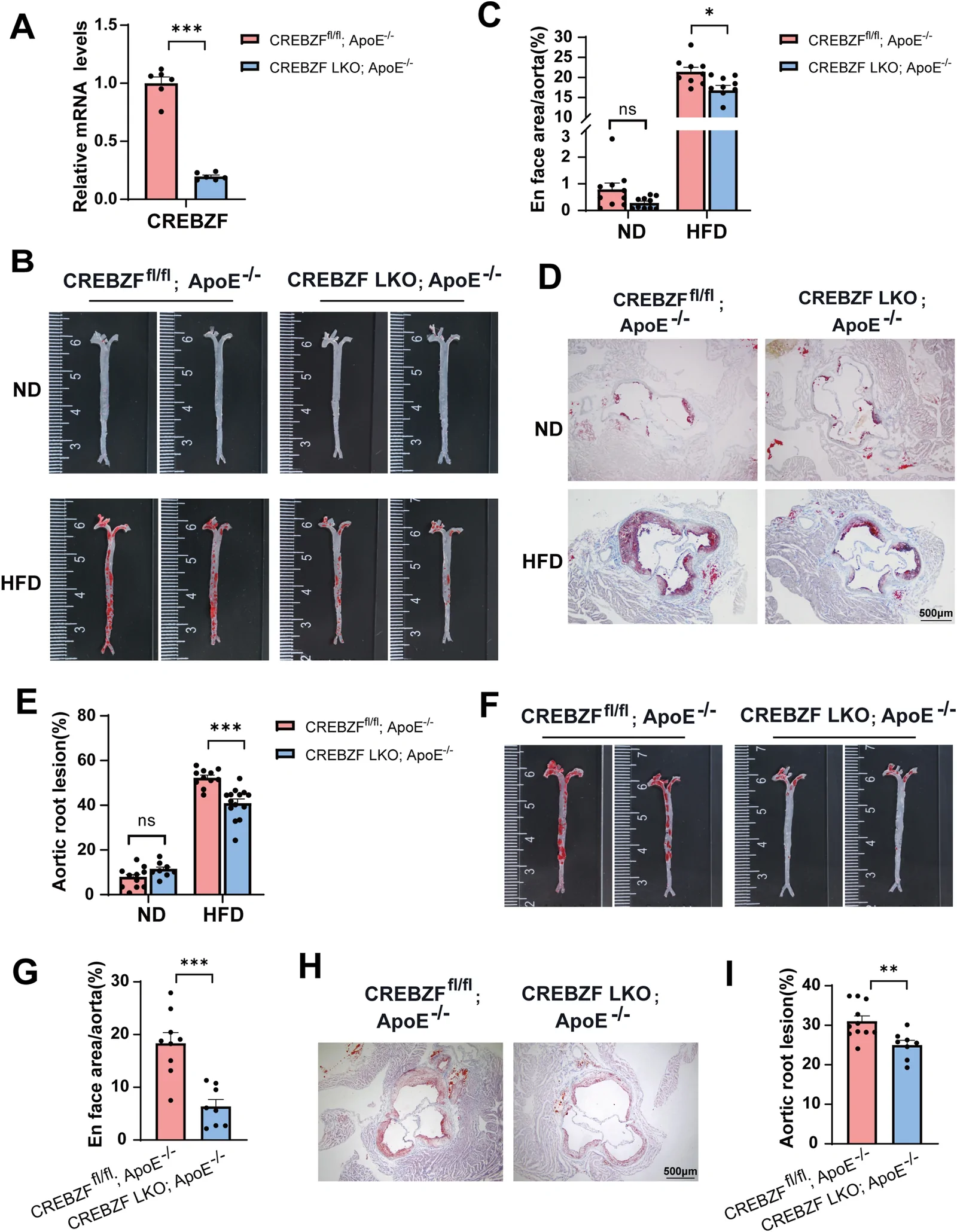
Hepatocyte-specific deletion of CREBZF attenuates atherosclerotic lesions. **(A)** The expression of CREBZF was analyzed by q-PCR in *CREBZF*^fl/fl^; *ApoE*^−/−^ and *CREBZF* LKO; *ApoE*^−/−^. The CREBZF knockout efficiency in the liver was approximately 84%. **(B)***CREBZF*^fl/fl^; *ApoE*^−/−^ and CREBZF LKO; *ApoE*^−/−^ mice (8 weeks old, male, *n* ≥ 8 mice per group) were fed a ND or HFD for 12 weeks. Aortic atherosclerotic plaques were visualized using ORO staining followed by en face microscopy. **(C)** The lesion areas in the whole aorta were analyzed. **(D)** Representative images of ORO-stained aortic root. **(E)** The plaque area in the aortic root was quantified. **(F)***CREBZF*^fl/fl^; *ApoE*^−/−^ and *CREBZF* LKO; *ApoE*^−/−^ mice (8 weeks old, male, *n* ≥ 8 mice per group) were fed ND for 12 months. Aortic plaques were detected by ORO staining and en face microscopy. **(G)** The lesion areas in the whole aorta were analyzed. **(H)** Representative photomicrographs of ORO-stained aortic root sections. **(I)** The plaque area in the aortic root was quantified. ∗*p* < 0.05, ∗∗*p* < 0.01, ∗∗∗*p* < 0.001.

### Loss of hepatic CREBZF attenuated endothelial inflammation at the aortic root in *ApoE*^−/−^ mice but did not improve lipid metabolism parameters

AS is a chronic arterial wall inflammation that is fundamentally driven by systemic metabolic and inflammatory cues.^24^ As a central metabolic organ, the liver significantly contributes to this systemic environment. The liver releases critical mediators, such as cholesterol and a spectrum of inflammatory secretory proteins, into the bloodstream.^25^^,^^26^ To further elucidate how hepatic CREBZF influences plaque formation, we quantified hepatic and circulating levels of cholesterol and triglycerides. However, no significant differences were detected between *CREBZF*^fl/fl^; *ApoE*^−/−^ and *CREBZF* LKO; *ApoE*^−/−^ mice fed HFD or ND (Fig. S3). These results suggest that hepatic CREBZF deficiency attenuates atherosclerotic development independent of improvements in lipid metabolism.

Endothelial inflammation serves as a central driver in the initiation and progression of atherosclerosis.^27^ To assess the impact of hepatic CREBZF on aortic inflammation, we examined the expression of inflammatory markers in aortic root frozen sections via immunofluorescence staining. HFD feeding for 12 weeks resulted in pronounced upregulation of endothelial ICAM1 and MCP1; however, this induction was significantly blunted in mice with hepatic CREBZF deficiency. As key effector molecules predominantly produced by activated ECs, MCP1 and ICAM1 play pivotal roles in initiating monocyte adhesion and subsequent transendothelial migration. Consistent with the reduced endothelial activation, immunostaining for the macrophage-specific marker CD68 demonstrated a significant reduction in macrophage infiltration within the atherosclerotic plaques of CREBZF-deficient mice (Fig. 2A and B). Furthermore, following 12 months of ND, MCP1 expression and macrophages in the lesion area were also markedly reduced (Fig. 2C and D). These results demonstrate that liver-specific deletion of CREBZF markedly reduces endothelial inflammation levels.

**Figure 2:**
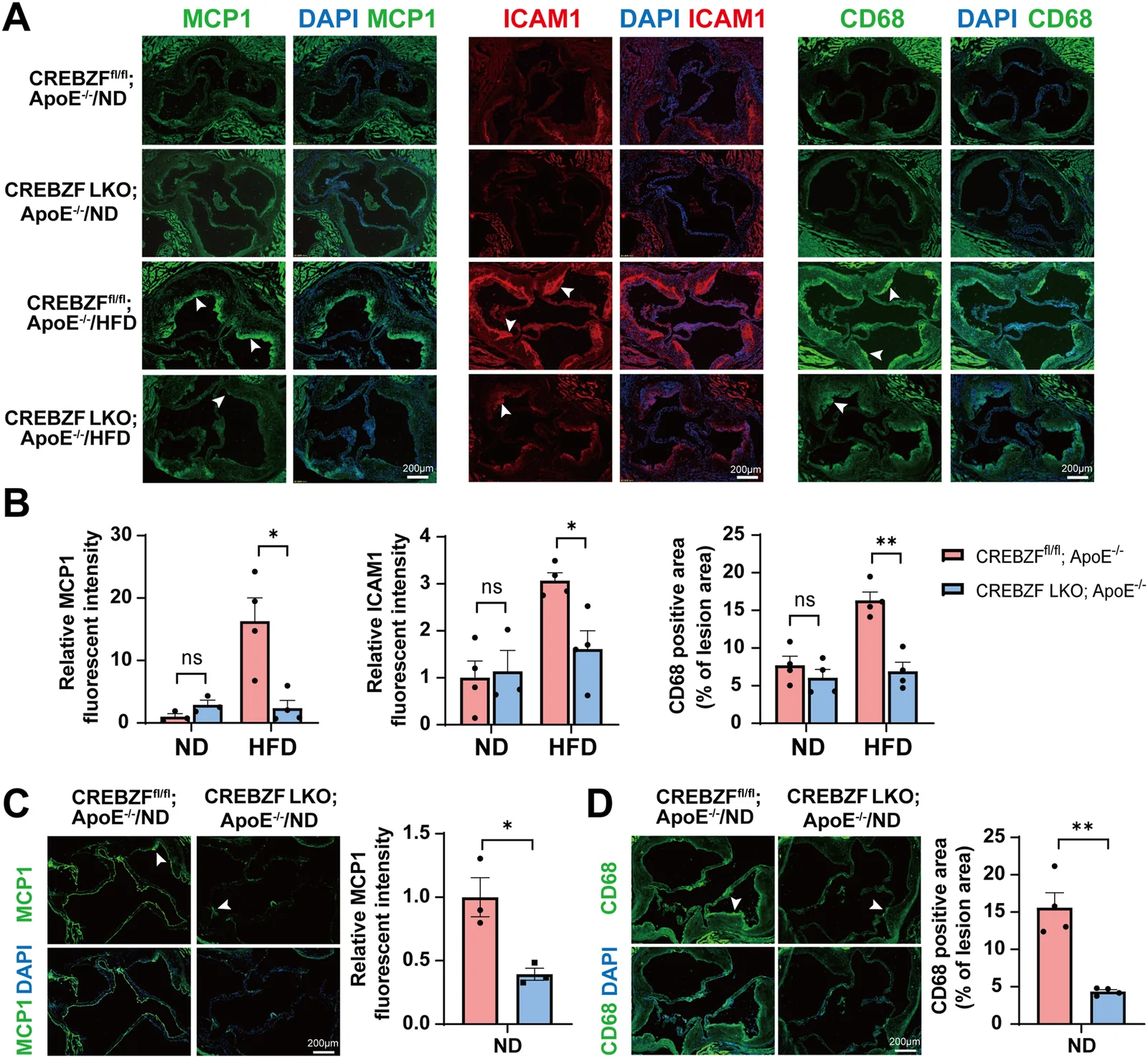
Hepatocyte-specific deletion of CREBZF ameliorates aortic plaque inflammation and mitigates macrophage infiltration. **(A, B)***CREBZF*^fl/fl^; *ApoE*^−/−^ and *CREBZF* LKO; *ApoE*^−/−^ mice (8 weeks old, male, *n* ≥ 3 mice per group) were fed ND or HFD for 12 weeks. (A) Immunofluorescence images of MCP1^+^ (green), ICAM1^+^ (red) and CD68^+^ (green) in *CREBZF*^fl/fl^; *ApoE*^−/−^ and *CREBZF* LKO; *ApoE*^−/−^ mice. DAPI, blue. Scale bars, 200 μm. (B) Quantitative analysis of MCP1, ICAM1 and CD68 in *CREBZF*^fl/fl^; *ApoE*^−/−^ and *CREBZF* LKO; *ApoE*^−/−^ mice. **(C, D)***CREBZF*^fl/fl^; *ApoE*^−/−^ and *CREBZF* LKO; *ApoE*^−/−^ mice (8 weeks old, male, *n* ≥ 3 mice per group) were fed ND for 12 months. (C) Immunofluorescence images and quantitative analysis of MCP1^+^ (green) in *CREBZF*^fl/fl^; *ApoE*^−/−^ and *CREBZF* LKO; *ApoE*^−/−^ mice. DAPI, blue. Scale bars, 200 μm. (D) Immunofluorescence images and quantitative analysis of CD68^+^ (green) in *CREBZF*^fl/fl^; *ApoE*^−/−^ and *CREBZF* LKO; *ApoE*^−/−^ mice. DAPI, blue. Scale bars, 200 μm. *n* ≥ 3 mice per group. ∗*p* < 0.05, ∗∗*p* < 0.01.

### Hepatocyte CREBZF modulates endothelial inflammation in a paracrine manner *in vitro*

To further confirm that hepatocyte CREBZF regulates endothelial inflammation, we performed gain- and loss-of-function experiments validated with HepG2 (with low endogenous CREBZF expression) and AML12 (with high endogenous CREBZF expression) cells. We successfully constructed a CREBZF-overexpressing adenovirus (Ad-CREBZF) and transfected it into HepG2 cells (Fig. S4A–S4C), and then analyzed the inflammatory status of the HepG2 cells themselves. Since NF-κB orchestrates inflammatory gene expression, we next asked whether CREBZF influences the NF-κB pathway. The results showed that CREBZF overexpression significantly increased the expression of phosphorylated p65 (p-p65) and VCAM1 in HepG2 cells (Fig. 3A and B). We subsequently knocked down CREBZF in AML12 cells via siRNA (Fig. S4D–S4F). The results revealed that CREBZF knockdown markedly reduced the expression of p-p65 in AML12 cells (Fig. 3C and D). To assess the role of hepatocyte-derived signals, we treated HUVECs with conditioned medium (CM) from hepatocytes with CREBZF overexpression or knockdown and then quantified the expression of inflammatory factors. The results demonstrated that CM from CREBZF-overexpressing HepG2 cells promoted the mRNA expression of MCP1, IL-6, and TNF-α, as well as the protein expression of ICAM1 and p-p65, in HUVECs (Fig. 3E–G). Conversely, CM from CREBZF-knockdown AML12 cells significantly reduced the mRNA levels of MCP1, IL-6, TNF-α, and IL-1β, as well as the protein expression of ICAM1 and p-p65, in HUVECs (Fig. 3H–J). These findings indicate that CREBZF in hepatocytes can dynamically regulate endothelial inflammation.

**Figure 3:**
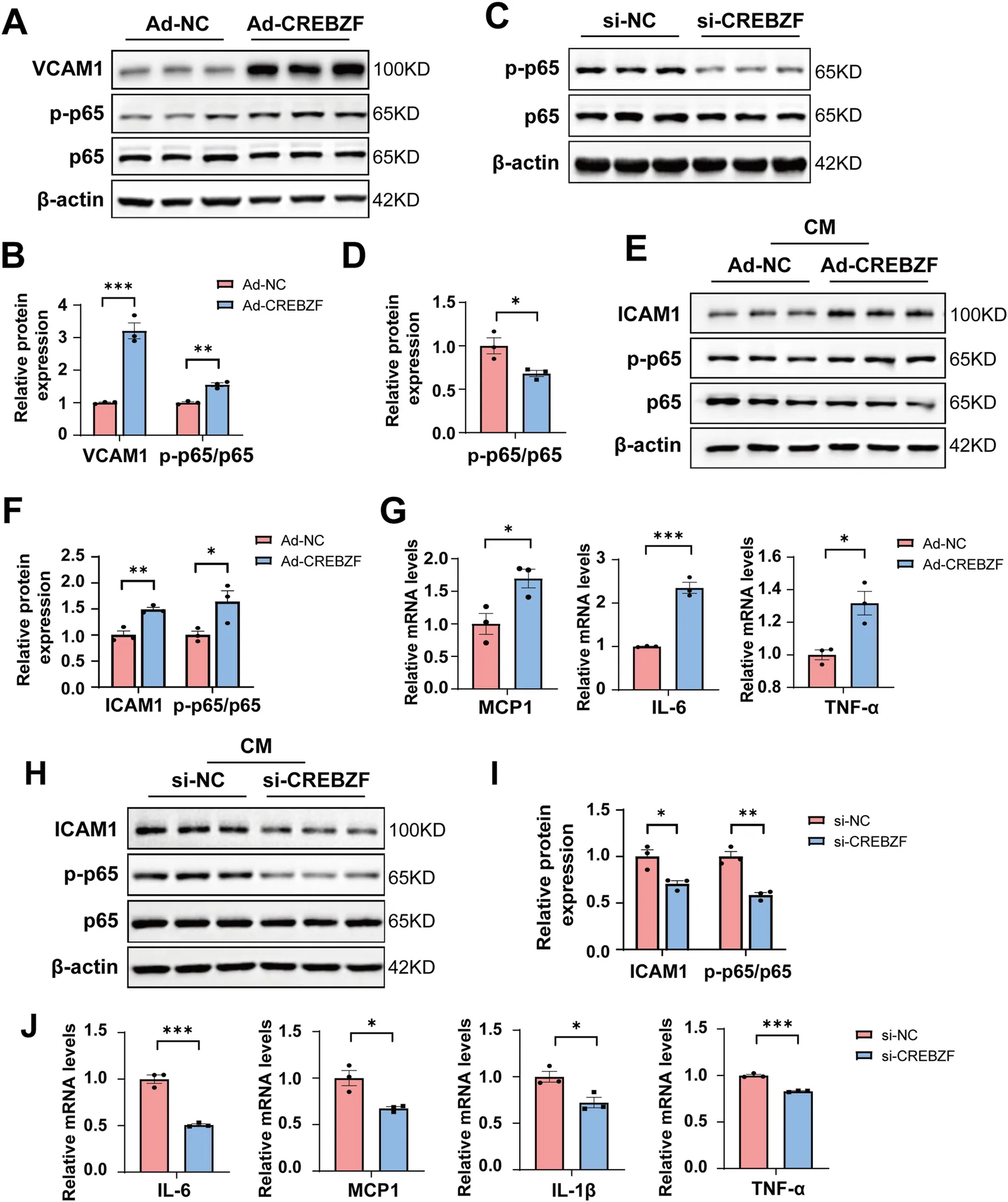
Hepatocyte CREBZF stimulates the inflammatory response in HUVECs. **(A)** Protein levels of VCAM1 and p-p65 in HepG2 cells. Ad-NC, control adenovirus. Ad-CREBZF, CREBZF-overexpressing adenovirus. **(B)** Quantification of VCAM1 and p-p65. **(C)** Protein levels of p-p65 in AML12. **(D)** Quantification of p-p65. **(E)** Protein levels of ICAM1 and p-p65 in HUVECs. CM, culture medium derived from HepG2 cells infected with control adenovirus or CREBZF-overexpressing adenovirus. **(F)** Quantification of ICAM1 and p-p65. **(G)** mRNA levels of MCP1, IL-6, and TNF-α in HUVECs stimulated with HepG2-derived culture medium. **(H)** Protein levels of ICAM1 and p-p65 in HUVECs stimulated with AML12-derived culture medium. si-NC, control siRNA. si-CREBZF, CREBZF-specific siRNA. CM, culture medium derived from AML12 infected with control siRNA or CREBZF siRNA. **(I)** Quantification of ICAM1 and p-p65. **(J)** mRNA levels of IL-6, MCP1, IL-1β, and TNF-α in HUVECs stimulated with AML12-derived culture medium. ∗*p* < 0.05, ∗∗*p* < 0.01, ∗∗∗*p* < 0.001.

### Hepatocyte CREBZF exacerbates endothelial inflammation through exosome-mediated mechanisms

The liver regulates the function of distal blood vessels through the secretion of soluble proteins and EVs, particularly exosomes. Given that systemic inflammation is a direct and critical driver of atherosclerotic progression, and that IL-1β, TNF-α, MCP1, and IL-6 are core pro-inflammatory cytokines mediating this pathological process, we sought to investigate whether hepatic CREBZF deficiency attenuates atherosclerosis by modulating systemic inflammatory status. To this end, we quantified the serum levels of IL-6, IL-1β, MCP1, and TNF-α by ELISA. However, no significant differences were observed between the groups (Fig. S4). Given that there are no significant changes in cholesterol or inflammatory factors in the serum, we shifted our focus to hepatocyte-derived exosome-nanoscale EVs that serve as potent vehicles for the targeted delivery of proteins, lipids, and nucleic acids. To isolate hepatocyte-derived exosomes, we performed differential ultracentrifugation of cell culture media. Nanoparticle tracking analysis (NTA) confirmed that the isolated particles had a size distribution of 30–150 nm and a characteristic concentration (Fig. 4A and B). To further determine whether hepatic CREBZF regulates exosome production, we examined the expression of genes involved in exosome biogenesis and secretion. The results showed that overexpression of CREBZF in hepatocytes did not significantly affect the process of exosome biogenesis (Fig. 4C). Their exosomal morphology was further verified by transmission electron microscopy (TEM) (Fig. 4D). Immunoblot analysis detected enrichment of the exosome-specific markers Tsg101, CD63, and CD81 (Fig. 4E), demonstrating that the majority of the isolated EVs were exosomes of hepatocyte origin.

**Figure 4:**
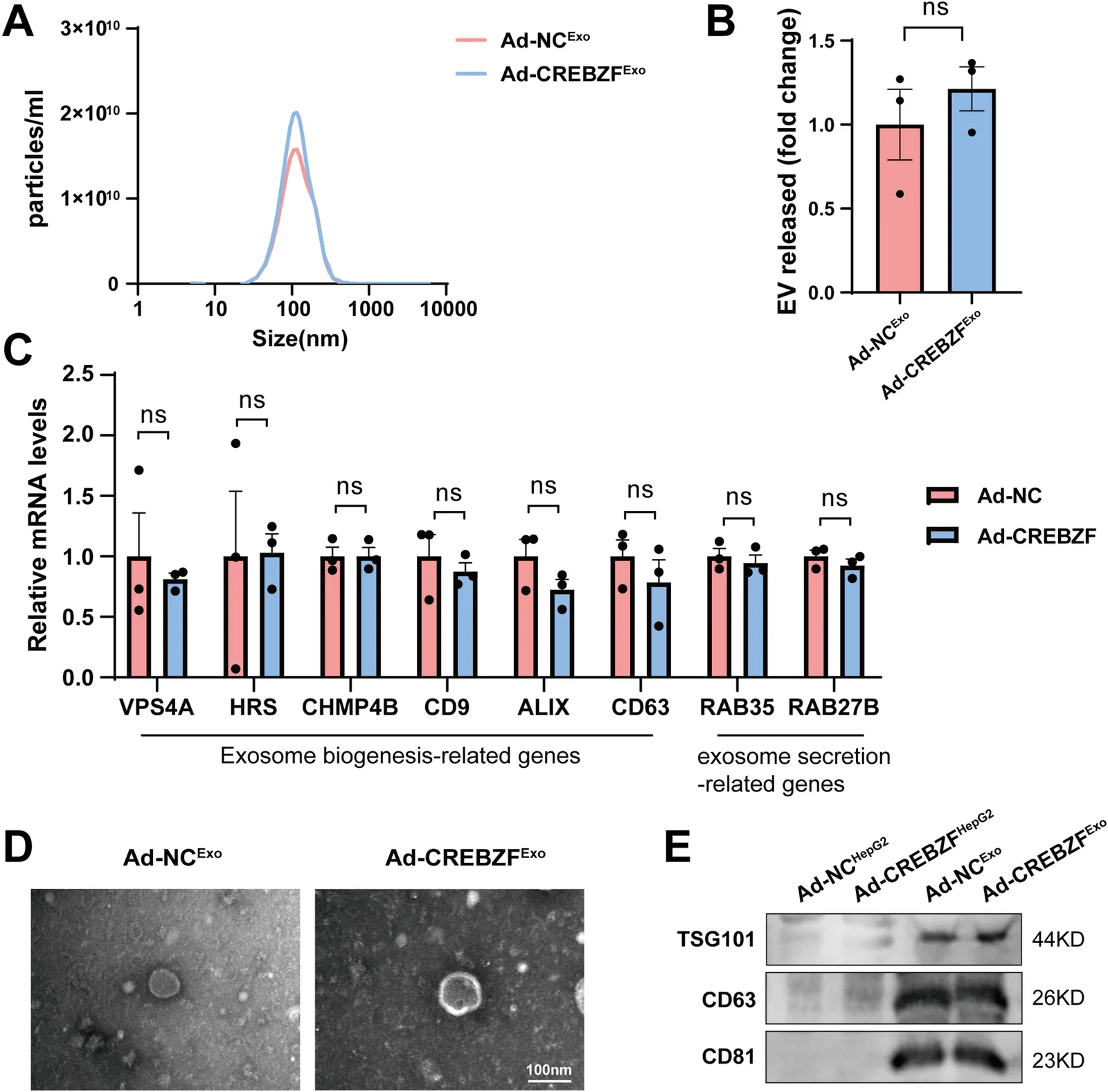
Characterization of hepatocyte-derived exosomes. HepG2 cells were treated with control adenovirus (Ad-NC) or CREBZF-overexpressing adenovirus (Ad-CREBZF) for 48 h, followed by replacement with exosome-depleted serum medium for an additional 24 h of culture. Exosomes were isolated from the conditioned medium using differential ultracentrifugation and normalized based on the total cell count (referred to as Ad-NC^Exo^ and Ad-CREBZF^Exo^, respectively). **(A)** The concentration and size distribution of exosomes were analyzed by NTA. **(B)** Quantification of exosome concentration. **(C)** Quantification of exosome biogenesis and secretion-related gene expression in hepatocytes infected with CREBZF-overexpressing adenovirus compared to control virus. **(D)** Representative TEM images of Ad-NC^Exo^ and Ad-CREBZF^Exo^, scale bar = 100 nm. **(E)** Representative Western blots showing the expression of exosomal markers (TSG101, CD63, and CD81) in total protein lysates from HepG2 cells and HepG2-derived exosomes.

To investigate the role of hepatocyte-derived exosomes in mediating hepatic-vascular communication, we first asked whether these exosomes could be internalized by ECs. Exosomes derived from HepG2 hepatocytes pre-treated with either CREBZF-overexpressing adenovirus (Ad-CREBZF^Exo^) or control adenovirus (Ad-NC^Exo^) were labeled with the fluorescent dye PKH67. Following a 12-h incubation with PKH67-labeled exosomes, distinct green fluorescence was detected within HUVECs (Fig. 5A), indicating efficient internalization of these hepatocyte-derived vesicles.

**Figure 5:**
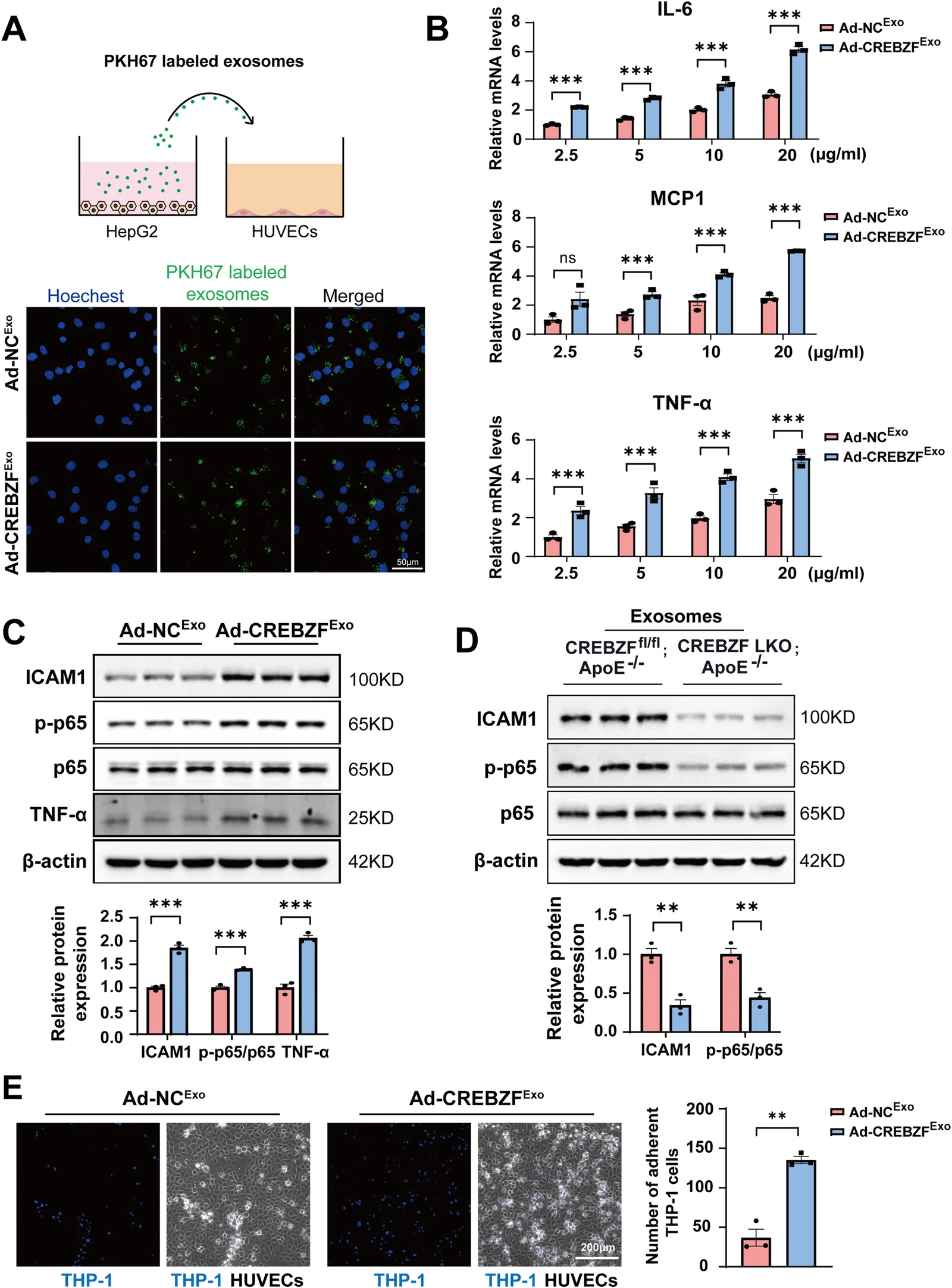
Hepatocyte CREBZF governs endothelial inflammation via exosomal signaling. Hepatocyte-derived exosomes were isolated from the HepG2 cells, which were treated with control adenovirus (Ad-NC) or CREBZF-overexpressing adenovirus (Ad-CREBZF). **(A)** Exosomes derived from Ad-NC- or Ad-CREBZF-treated HepG2 cells (Ad-NC^Exo^ or Ad-CREBZF^Exo^) were labelled with PKH67 fluorescent dye and subsequently co-incubated with HUVECs for 12 h. Representative fluorescent micrographs showing PKH67-labeled exosomes (green) within HUVECs. **(B)** Relative mRNA levels of IL-6, MCP1 and TNF-α were determined in HUVECs that were co-cultured with Ad-NC^Exo^ or Ad-CREBZF^Exo^ at different concentrations. **(C)** Representative Western blots and quantification analysis showing the expression of ICAM1, p-p65 and TNF-α in HUVECs following treatment with Ad-NC^Exo^ or Ad-CREBZF^Exo^. **(D)** Representative Western blots and quantification analysis showing the expression of ICAM1 and p-p65 in HUVECs following treatment with *CREBZF*^fl/fl^; *ApoE*^−/−Exo^ or *CREBZF* LKO; *ApoE*^−/−Exo^. **(E)** Representative images and quantitative analysis of THP-1 adhesion to HUVECs. THP-1 labeled with Hoechst, blue; HUVECs, brightfield. Scale bars, 200 μm. ∗∗*p* < 0.01, ∗∗∗*p* < 0.001.

Having established that ECs internalize hepatocyte-derived exosomes, we next asked how these vesicles affect recipient cell function. Accordingly, HUVECs were treated with Ad-NC^Exo^ or Ad-CREBZF^Exo^ at various concentrations, and the inflammatory response was then analyzed via q-PCR. As shown in Figure 5B, the hepatocyte-derived exosomes induced the upregulation of proinflammatory molecules (IL-6, MCP1, and TNF-α). Furthermore, a stronger inflammatory response was observed in the Ad-CREBZF^Exo^-treated ECs relative to the control group. Consistent with the above findings, Ad-CREBZF^Exo^ treatment resulted in significant increases in TNF-α, ICAM1, and p-p65 protein expression in ECs compared with Ad-NC^Exo^-treated cells (Fig. 5C). To determine whether factors other than exosomes contribute to endothelial inflammation, we collected culture medium from hepatocytes overexpressing CREBZF or control hepatocytes, depleted it from exosomes, and then applied it to HUVECs. The results showed that exosome-depleted hepatocyte culture medium had no significant effect on endothelial inflammation (Fig. S6). Hepatic exosomes exert their effects on ECs via the circulatory system. To determine whether our *in vitro* and *in vivo* observations were concordant, we treated HUVECs with serum exosomes isolated from *CREBZF*^fl/fl^; *ApoE*^−/−^ and *CREBZF* LKO; *ApoE*^−/−^ mice. The results demonstrated that exosomes from *CREBZF* LKO mice markedly attenuated the protein expression of ICAM1 and phospho-p65 in ECs (Fig. 5D). Elevated endothelial inflammation recruits monocyte accumulation, a key initiating event in atherogenesis. Using a co-culture system of THP-1 cells and HUVECs, we demonstrated that exosomes derived from CREBZF-overexpressing hepatocytes significantly enhanced the adhesive capacity of HUVECs to THP-1 cells (Fig. 5E). These results demonstrate that hepatocyte CREBZF participates in the progression of atherosclerosis through exosome-mediated regulation of endothelial inflammation.

### Differential miRNA expression profile in CREBZF-overexpressing hepatocyte-derived exosomes

To determine whether CREBZF alters the composition of exosomes, we employed Illumina miRNA deep-sequencing to perform a comprehensive comparison of the miRNA expression profiles between exosomes derived from CREBZF-overexpressing and control adenovirus-treated HepG2 hepatocytes, aiming to identify mediators of their proinflammatory effects. Analysis of the sequencing data revealed substantial differences in exosomal miRNA expression between the two groups. The 13 miRNAs with pronounced significant differences (fold change > 2) are illustrated in the heatmap shown in Figure 6A. Among them, five miRNAs (miR-210-3p, miR-1274-5p, miR-185-3p, miR-4750-3p, and miR-29a-3p) were highly expressed in HepG2 cells. Evidence has shown that the expression of miR-29a-3p or miR-210-3p is downregulated in response to inflammation in animals.^28^^,^^29^ The decreased miR-29a-3p and miR-210-3p expression in CREBZF-overexpressing hepatocyte-derived EVs was confirmed by q-PCR analysis (Fig. 6B). Consistently, reduced levels of these two miRNAs were also detected in Ad-CREBZF^Exo^-treated ECs (Fig. 6C). In contrast, genetic deletion of CREBZF in hepatocytes resulted in a marked increase in the levels of miR-29a-3p and miR-210-3p in circulating serum exosomes, as observed relative to those in CREBZF^fl/fl^; *ApoE*^−/−^ controls (Fig. 6D). Thus, we chose miR-29a-3p and miR-210-3p as candidate miRNAs that potentially regulate endothelial inflammation.

**Figure 6:**
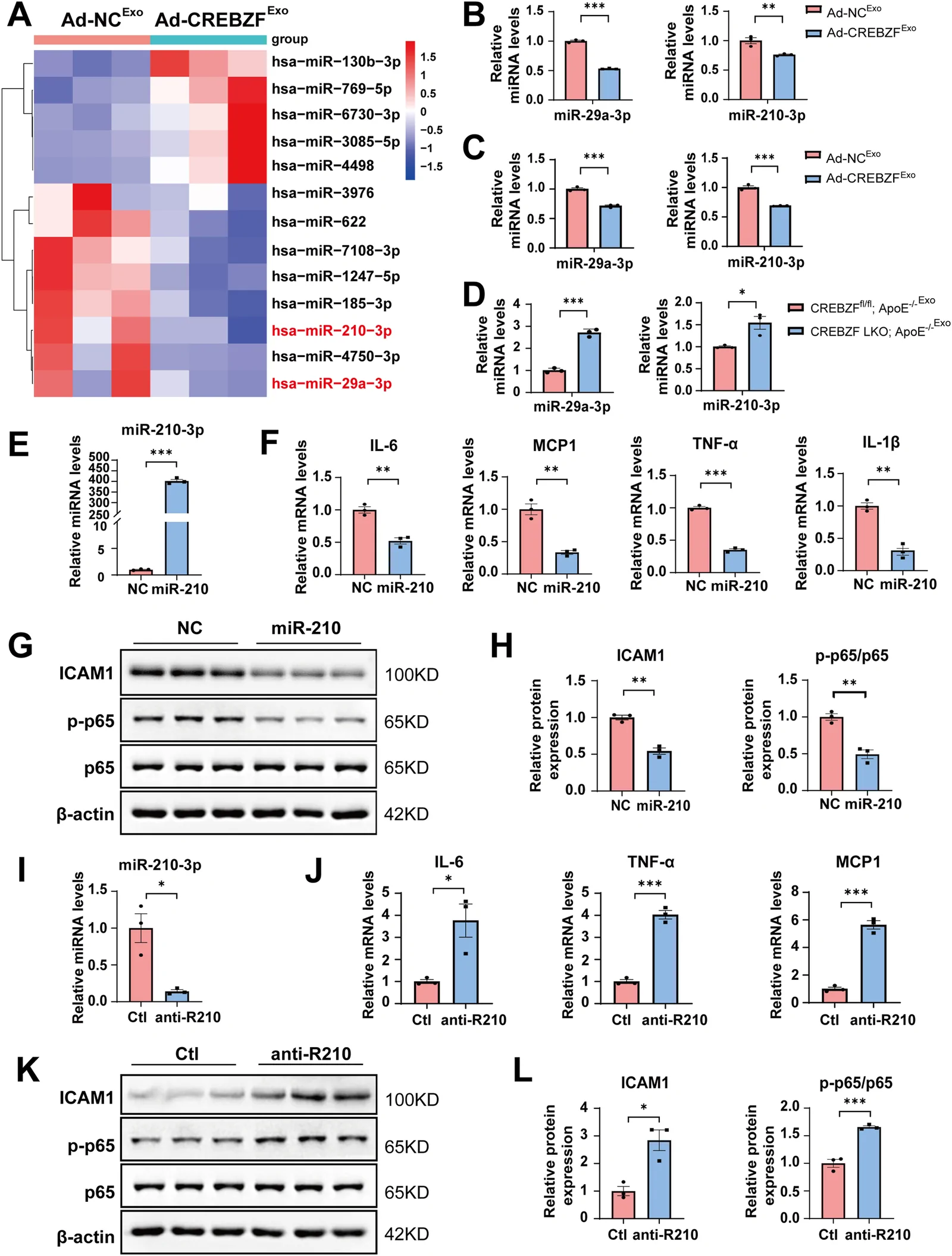
Expression profile of miRNAs in hepatocyte-derived exosomes and anti-inflammatory effect of miR-210-3p on HUVECs. **(A)** The heatmap depicts the 13 differentially expressed miRNAs identified in exosomes derived from HepG2 cells treated with either Ad-NC or Ad-CREBZF. **(B)** The levels of miR-29a-3p and miR-210-3p in exosomes derived from HepG2 cells treated with either Ad-NC or Ad-CREBZF (referred to as Ad-NC^Exo^ and Ad-CREBZF^Exo^, respectively) were analyzed by q-PCR. **(C)** The levels of miR-29a-3p and miR-210-3p in HUVECs following treatment with Ad-NC^Exo^ or Ad-CREBZF^Exo^ were analyzed by q-PCR. **(D)** q-PCR analysis of miR-29a-3p and miR-210-3p expression in serum exosomes from *CREBZF*^fl/fl^; *ApoE*^−/−Exo^ or *CREBZF* LKO; *ApoE*^−/−Exo^ mice. **(E, F)** Following miR-210-3p mimic treatment, the levels of miR-210-3p, IL-6, MCP1, TNF-α, and IL-1β were determined by q-PCR. **(G, H)** The relative fold changes in the ICAM1 and p-p65 protein levels following miR-210-3p mimic treatment. **(I, J)** Following miR-210-3p inhibitor treatment, the levels of miR-210-3p, IL-6, MCP1, and TNF-α were determined via q-PCR. **(K, L)** The relative fold changes in the ICAM1 and p-p65 protein levels following miR-210-3p inhibitor treatment. ∗*p* < 0.05, ∗∗*p* < 0.01, ∗∗∗*p* < 0.001.

### miR-210-3p suppresses endothelial inflammation through targeted inhibition of C-Rel

The candidate miRNAs miR-29a-3p and miR-210-3p were selected for functional validation. Each was overexpressed in HUVECs to assess its impact on inflammatory responses. The level of miR-29a-3p increased approximately 14-fold, whereas that of miR-210-3p increased approximately 400-fold with the same concentrations of miRNA mimic (Fig. S7A; Fig. 6E). We next examined the effects of miR-29a-3p and miR-210-3p on key inflammatory factors. Overexpression of miR-210-3p in HUVECs resulted in a 2- to 3-fold decrease in the expression of proinflammatory genes (IL-6, MCP1, TNF-α, and IL-1β) (Fig. 6F). However, in HUVECs overexpressing miR-29a-3p, only MCP1 and IL-6 were reduced, while TNF-α was increased (Fig. S7B). Notably, miR-210-3p significantly decreased the protein expression of ICAM1 and p-p65 (Fig. 6G and H). In contrast, miR-29a-3p had no significant effect on ICAM1 or p-p65 (Fig. S7C and S7D). To further confirm the anti-inflammatory capacity of miR-29a-3p and miR-210-3p, we treated HUVECs with their respective inhibitors and measured the expression of key inflammatory factors. The level of miR-29a-3p decreased approximately 80%, whereas that of miR-210-3p decreased approximately 86% with the same concentrations of miRNA inhibitor (Fig. S7E; Fig. 6I). These results indicate that only miR-210-3p is a potent anti-inflammatory factor in HUVECs (Fig. S7F–S7H; Fig. 6J–L). These findings suggest a mechanism whereby hepatocyte CREBZF regulates endothelial inflammation through exosomal miR-210-3p.

We did not identify any predicted binding sites for miR-210-3p within the mRNA sequences of adhesion molecules such as ICAM1 and VCAM1, nor in inflammatory cytokines, including IL-6 and TNF-α. Consequently, we directed our attention to the NF-κB family, a master regulator of inflammatory responses. Upon stimulation by pathogens or cytokines such as TNF-α and IL-1β, NF-κB rapidly translocates into the nucleus to activate downstream target gene expression. Bioinformatics analysis revealed a putative miR-210-3p binding site within the 3′ UTR of C-Rel, a key member of the NF-κB transcription factor family (Fig. 7A). Subsequent experiments showed that treatment of HUVECs with miR-210-3p significantly reduced C-Rel protein expression (Fig. 7B). To validate whether C-Rel is a direct target of miR-210-3p, we performed a luciferase reporter assay. Co-transfection of miR-210-3p mimics with a C-Rel 3′-UTR reporter construct into HEK293T cells markedly suppressed luciferase activity, an effect that was abrogated upon mutation of the miR-210-3p binding site (Fig. 7C). Furthermore, overexpression of C-Rel in HUVECs completely reversed the anti-inflammatory effects of miR-210-3p (Fig. 7D). Collectively, these results demonstrate that miR-210-3p suppresses downstream inflammatory pathways by directly targeting C-Rel.

**Figure 7:**
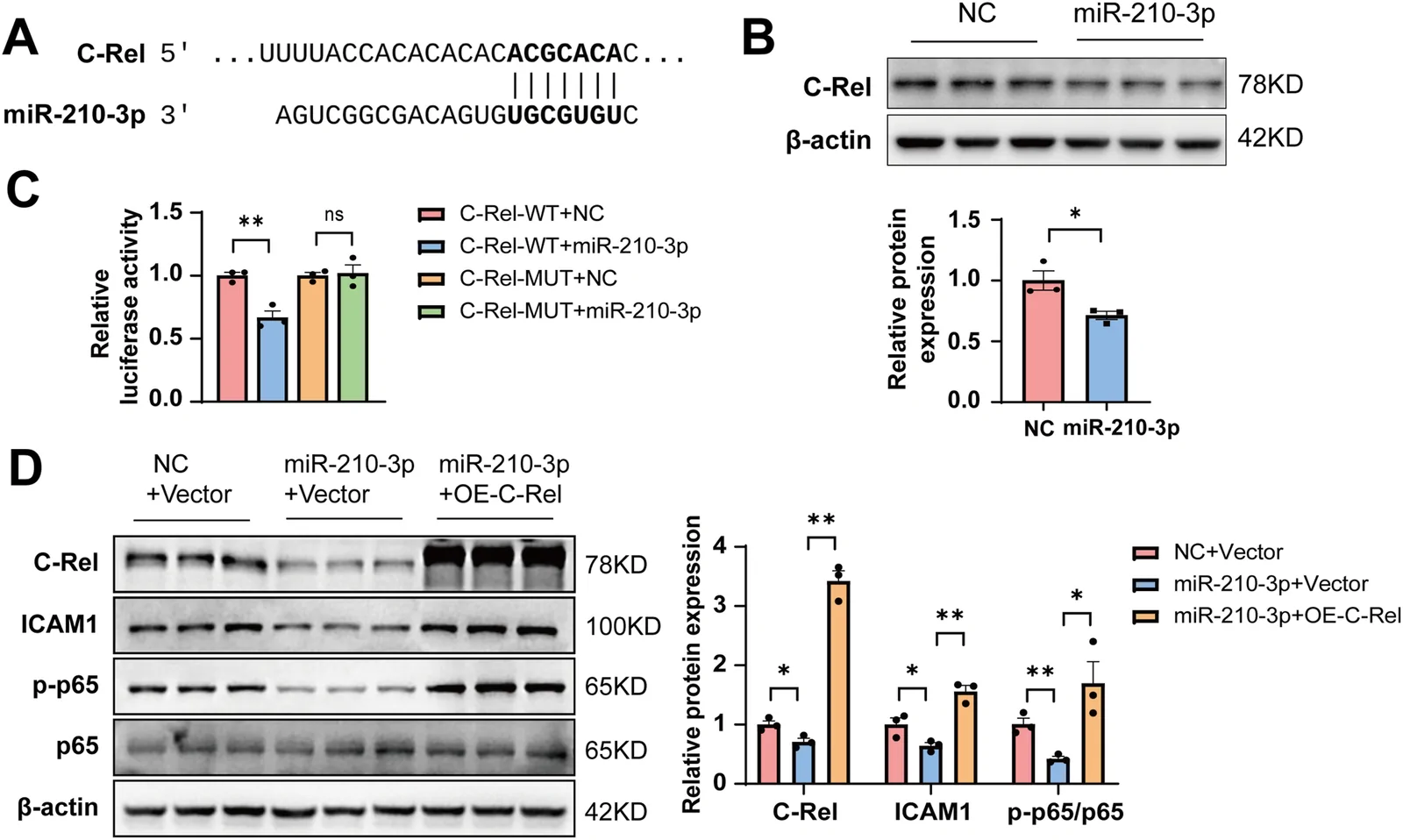
miR-210-3p attenuates endothelial inflammation through direct binding to C-Rel. **(A)** The predicted binding sites of miR-210-3p within the 3′ UTR of the human C-Rel gene. **(B)** C-Rel protein expression in HUVECs after treatment with miR-210-3p mimics. **(C)** HEK293T cells were co-transfected with the C-Rel 3′UTR reporter plasmid, a *Renilla* luciferase control plasmid, and either miR-210-3p mimic or negative control mimic. C-Rel reporter gene activity was measured and normalized to relative luciferase activity. **(D)** Relative fold changes in the protein levels of C-Rel, ICAM1, and p-p65. ∗*p* < 0.05, ∗∗*p* < 0.01.

### The miR-210-3p mimic rescued the endothelial inflammation stimulated by exosomes derived from CREBZF-overexpressing hepatocytes

Based on the anti-inflammatory effect of miR-210-3p, we proposed that its exosomal transfer from hepatocytes mediates CREBZF-driven endothelial inflammation. To verify this, we tested miRNA delivery by transfecting HepG2 cells with a Cy3-labeled miR-210-3p mimic and co-culturing them with HUVECs. Clear intracellular Cy3 accumulation in HUVECs after 24 h confirmed effective intercellular transport (Fig. 8A and B).

**Figure 8:**
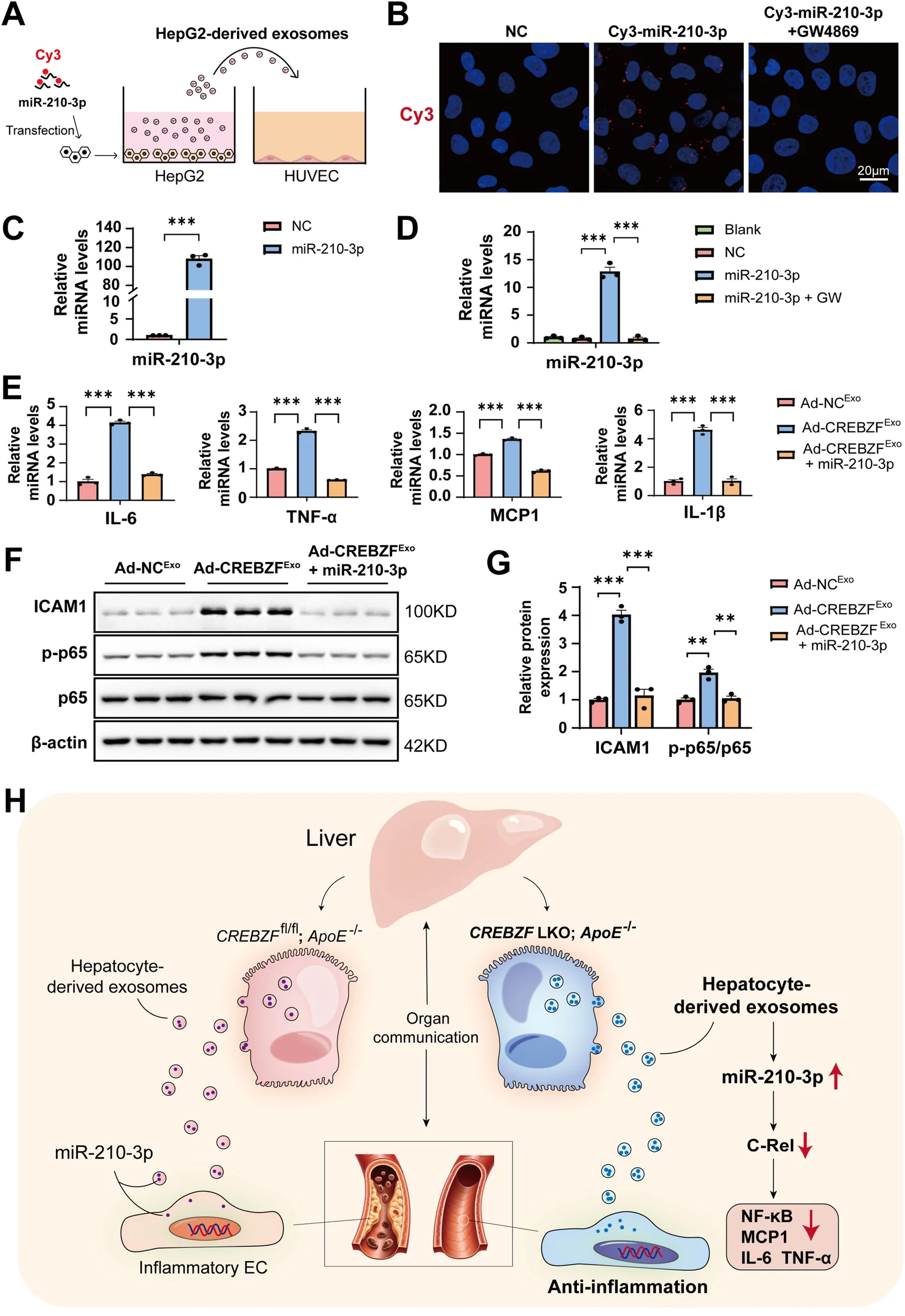
Effect of hepatocyte-derived exosomal miR-210-3p on HUVECs. **(A**–**D)** HepG2 cells were transfected with a Cy3-labeled miR-210-3p mimic or negative control, followed by treatment with or without the exosome secretion inhibitor GW4869 (20 μM). Conditioned media from the HepG2 cells were then collected and used to treat HUVECs for 24 h prior to analysis. (A) Schematic of the experimental procedure. (B) Fluorescent micrographs show Cy3-labeled miR-210-3p (red) inside HUVECs. (C) miR-210-3p expression in HepG2 cells was determined by q-PCR. (D) HUVECs were incubated with conditioned media from control or miR-210-3p-transfected HepG2 cells, with or without GW4869. miR-210-3p levels in HUVECs were then measured by q-PCR. **(E**–**G)** HUVECs were treated with Ad-NC^Exo^ or Ad-CREBZF^Exo^ isolated from HepG2 cells in the presence or absence of the miR-210-3p mimic, after which the cellular mRNA or protein levels were measured. (E) Relative fold changes in the levels of the IL-6, MCP1, TNF-α, and IL-1β mRNAs. (F, G) Relative fold changes in the levels of the ICAM1 and p-p65 proteins. **(H)** Schematic model depicting how hepatic CREBZF deficiency ameliorates plaque formation via upregulation of exosome-transported miR-210-3p. In *ApoE*^−/−^ background mice, both 12-week HFD and 12-month ND feeding successfully induced atherosclerotic formation. Liver-specific deletion of the transcription factor CREBZF significantly ameliorated plaque lesions. Mechanistically, CREBZF deficiency in hepatocytes promoted the upregulation of exosome-transported miR-210-3p. Upon uptake by recipient ECs, these hepatocyte-derived exosomes further increase intracellular miR-210-3p levels, leading to the suppression of key endothelial inflammatory factors and ultimately conferring atheroprotection. ∗∗*p*< 0.01, ∗∗∗*p* < 0.001.

After the hepatocytes were transfected with miR-210-3p mimics, we treated HUVECs with hepatocyte medium. Quantitative analysis revealed a substantial increase in miR-210-3p in HUVECs. When exosome release from hepatocytes was inhibited by GW4869, the increase in miR-210-3p in HUVECs was completely prevented, thus demonstrating that the transfer of miR-210-3p from hepatocytes to ECs is exosome-dependent (Fig. 8C and D). To confirm that CREBZF-driven hepatocyte exosomes regulate endothelial inflammation in a miR-210-3p-dependent manner, we treated HUVECs with exosomes derived from CREBZF-overexpressing or control adenovirus-treated hepatocytes (Ad-CREBZF^Exo^ or Ad-NC^Exo^), along with miR-210-3p mimics. Both the q-PCR and protein results demonstrated that miR-210-3p completely reversed the endothelial inflammation induced by exosomes from CREBZF-overexpressing hepatocytes (Fig. 8E–G). In summary, these results demonstrate that miR-210-3p is critical for the regulation of endothelial inflammation by CREBZF-driven hepatocyte exosomes.

## Discussion

Prevailing understanding of atherosclerosis, substantiated by our prior work and that of others, has largely maintained a “vascular-centric” perspective.^30^, ^31^, ^32^ This paradigm has productively focused on local lipid deposition and the phenotypic modulation of vascular cells within the arterial wall.^31^^,^^33^, ^34^, ^35^ However, this framework has predominantly overlooked the potential for systemic pathological regulation by remote organs. Our current work significantly expands this conventional view by establishing the liver as a pivotal upstream regulator of atherogenesis.

Herein, we provide direct experimental evidence demonstrating that targeted deletion of hepatic CREBZF attenuates atherosclerotic progression, underscoring its therapeutic potential. Mechanistically, we propose a signaling paradigm, the miR-210-3p/NF-κB axis, through which hepatocyte-derived exosomes promote endothelial inflammation and atherogenesis. Collectively, these findings reveal the crucial role of CREBZF-guided exosomal communication in liver-vascular crosstalk. Moreover, they uncover a previously unrecognized mechanistic link between hepatic signaling and extrahepatic vascular pathogenesis (Fig. 8H).

Abnormal liver function has been widely recognized as an independent risk factor for atherosclerotic cardiovascular disease. Studies have shown that hepatic CREBZF deletion ameliorates hepatocyte inflammation and lipid accumulation.^36^^,^^37^ To investigate the vascular regulatory role of hepatic CREBZF, we generated liver-specific CREBZF knockout mice on an *ApoE*^−/−^ background. Consistent with previous studies, CREBZF knockout alleviated hepatocyte inflammation.^20^ However, our study revealed a discrepant finding: *CREBZF* LKO; *ApoE*^−/−^ mice presented no significant decrease in hepatic lipid accumulation. We propose that the profound lipid homeostatic imbalance caused by ApoE deletion likely overwhelms the regulatory role of CREBZF in hepatic lipid metabolism.^38^ ApoE-mediated lipoprotein clearance regulates cholesterol homeostasis and triglyceride metabolism.^39^ Coupled with our findings of similar hepatic and serum cholesterol/triglyceride profiles in *CREBZF* LKO; *ApoE*^−/−^ versus control mice (Fig. S3), we further hypothesize that the lipid-regulatory function of CREBZF may be dependent on the ApoE-mediated lipoprotein clearance pathway. Strikingly, hepatic CREBZF deficiency ameliorates atherosclerotic plaques through attenuation of endothelial inflammation (Figure 1, Figure 2, Figure 3). To investigate the underlying mechanism, we further examined the levels of inflammatory factors in the serum (Fig. S5). Our data indicate that hepatic CREBZF deficiency does not significantly alter the serum levels of soluble inflammatory factors. This finding is entirely plausible for the following reasons: Soluble inflammatory cytokines such as TNF-α and IL-6 are primarily secreted into the bloodstream by immune cells. In contrast, exosomes derived from hepatocytes do not directly contribute to the circulating pool of these proteins. Upon uptake by ECs, exosomal miRNAs modulate inflammatory signaling pathways within the endothelium, thereby influencing monocyte adhesion—a process localized to the vascular wall. Therefore, hepatic CREBZF deficiency regulates endothelial inflammation via exosomes without exerting a significant impact on systemic inflammation.

Research has demonstrated that liver-vascular communication occurs not only through direct contact and soluble cytokines (TNF-α, IL-6, IL-8, and others) but also via exosomes.^12^^,^^14^^,^^40^, ^41^, ^42^ We have unequivocally demonstrated that under varying CREBZF expression conditions, hepatocyte-derived exosomes act as signaling mediators that promote atherogenesis by regulating cellular inflammation. This notion is supported by Figure 4, Figure 5. Initially, using PKH67-labelled exosomes, we confirmed that HUVECs efficiently internalized hepatocyte-derived exosomes, a phenomenon that is consistent with previous studies.^15^^,^^43^ Moreover, a bidirectional regulatory effect was observed: *CREBZF* LKO; *ApoE*^−/−^ serum exosomes suppressed endothelial inflammation, whereas exosomes from CREBZF-overexpressing hepatocytes potently stimulated proinflammatory activation in ECs. Collectively, our data identify hepatic CREBZF as a key regulator that drives proinflammatory signaling to ECs via exosome-mediated communication.

Among the diverse bioactive cargo of exosomes, miRNAs have emerged as critical mediators of intercellular communication.^44^^,^^45^ Through analysis of miRNAs in hepatocyte-derived exosomes, we found that CREBZF overexpression in hepatocytes significantly altered the miRNA expression profile compared with control cells (Fig. 6). Among the 13 significantly altered miRNAs, we focused on five that exhibited relatively high abundances in hepatocytes. miR-29a-3p has been shown to alleviate hepatic inflammation and fibrosis by targeting MCJ and modulating the Hippo signaling pathway.^46^ Accumulating evidence from multiple studies has demonstrated that loss of miR-29a-3p exacerbates disease progression in multiple pathological contexts, such as acute pancreatitis, acute liver failure, and osteoarthritis.^47^, ^48^, ^49^ In addition, studies have demonstrated that miR-210-3p is induced by hypoxic conditions in the tumor microenvironment, where it promotes tumor progression by regulating cancer cell proliferation, angiogenesis, and metastasis while also exhibiting significant crosstalk with inflammatory responses.^50^, ^51^, ^52^ However, whether miR-29a-3p and miR-210-3p exert specific functions in ECs within the context of cardiometabolic disease has not been investigated. Owing to the practical impossibility of isolating exosomes from primary hepatocyte cultures, we alternatively isolated serum exosomes from *CREBZF* LKO; *ApoE*^−/−^ mice, which conversely confirmed that CREBZF knockdown led to significant upregulation of miR-210-3p and miR-29a-3p *in vivo* (Fig. 6D). In the present study, we identified that miR-210-3p, but not miR-29a-3p, has significant anti-inflammatory effects on ECs (Fig. 6; Fig. S7). Notably, while endogenous miR-210-3p is barely detectable in resting HUVECs, its levels were markedly elevated following treatment with exosomes from CREBZF-overexpressing hepatocytes. GW4869-mediated blockade of hepatocyte exosome release prevented the accumulation of miR-210-3p in ECs, confirming that hepatocytes deliver miR-210-3p via exosomes. Furthermore, the miR-210-3p mimic completely reversed the proinflammatory effects induced by CREBZF-overexpressing hepatocyte-derived exosomes on ECs (Fig. 8).

miRNAs function primarily as post-transcriptional regulators of gene expression. Bioinformatics analysis identified a putative miR-210-3p binding site within the 3′-untranslated region (3′-UTR) of C-Rel, a pivotal member of the NF-κB transcription factor family. We subsequently demonstrated that miR-210-3p overexpression significantly suppressed C-Rel protein expression. Luciferase reporter assays confirmed a direct interaction between miR-210-3p and the C-Rel 3′-UTR, and reintroduction of C-Rel in HUVECs completely abolished the anti-inflammatory effects of miR-210-3p (Fig. 7). Collectively, these findings establish the miR-210-3p/C-Rel regulatory axis as a critical mediator of hepatocyte-derived exosome-induced vascular endothelial inflammation. However, as CREBZF is a transcriptional cofactor that does not directly bind to miRNAs, the intermediate regulatory mechanisms remain to be fully elucidated. To further elucidate the pathophysiological relevance of miR-210-3p in endothelial inflammation and its broader implications in cardiometabolic disease, clinical assessment of its circulating levels in patients with atherosclerosis is warranted. Future investigations should systematically delineate the role of miR-210-3p in atherogenesis and its underlying molecular mechanisms.

In summary, our work establishes that hepatocyte-derived exosomes serve as critical mediators of liver-vascular communication. We further identified CREBZF as a key regulator controlling exosomal miRNA-mediated endothelial inflammation and plaque formation. The newly discovered anti-inflammatory factor miR-210-3p may represent a potential therapeutic target for preventing and treating cardiovascular diseases.

## CRediT authorship contribution statement

**Jinxuan Wang:** Writing – original draft, Validation, Methodology, Formal analysis. **Xiaoqi Zhou:** Methodology, Investigation, Formal analysis, Data curation. **Yuanyuan Liu:** Methodology, Investigation, Data curation. **Jianxiong Xu:** Writing – review & editing, Formal analysis. **Yanzhu Long:** Project administration. **Shuai Hu:** Methodology. **Cheng Wang:** Methodology. **Liqiong Yan:** Methodology. **Chen Yang:** Methodology. **Langtao Wang:** Project administration, Methodology. **Liping Luo:** Validation. **Tao Yu:** Methodology. **Yang Shen:** Supervision. **Yu Li:** Supervision. **Xiangyu Zhou:** Supervision. **Guixue Wang:** Writing – review & editing, Project administration. **Xiaozhen Dai:** Writing – review & editing, Supervision, Methodology, Investigation, Funding acquisition.

## Data availability

All data generated or analyzed during this study will be shared by the corresponding authors upon reasonable request.

## Funding

This work was supported by the National Key Technology R&D Program of China (No. 2023YFB3810100), the 10.13039/501100001809National Natural Science Foundation of China (No. 82470485, 82400488, 82170420), the 10.13039/501100018542Natural Science Foundation of Sichuan (China) (No. 2024NSFSC1615), the 10.13039/501100016350Sichuan Provincial Administration of Traditional Chinese Medicine (China) (No. 2024zd028), the Basic Medicine Research Innovation Center for Cardiometabolic Diseases, Ministry of Education Open Projects Fund (China) (No. xnykdxcxzx-2024-03), the Chengdu Medical College Science and Technology Innovation Team (China) (No. 2024kjTzn07), and the Organized Research Initiatives of Chengdu Medical College (China) (No. CYYZZ25-03).

## Conflict of interests

Dr. Guixue Wang is a member of *Genes & Diseases* Editorial Board. To minimize bias, Dr. Guixue Wang was excluded from all editorial decision-making related to the acceptance of this article for publication. The remaining authors declare no conflict of interests.
