# Supplementary material for: Targeting hepatic CREBZF protects against atherosclerosis via exosomal miR-210-3p mediated inhibition of endothelial inflammation
Source: Genes Dis. 2026 Apr 22;14(2):102212. doi: 10.1016/j.gendis.2026.102212 (PMC13677596; doi:10.1016/j.gendis.2026.102212)
Supplement: Supplementary file 1 [file mmc1.docx]

**Supplementary materials**

**Table S1** Primer sequences for PCR.

| **Target** | **Primer sequences (5’-3’)** |
| --- | --- |
| Mouse-CREBZF | F: 5’-GCCCGTCTTAATCGGCTCAA-3’ |
|  | R: 5’-CCGTAGGTAGCGACTCTCCTC-3’ |
| Human-CREBZF | F: 5’-GACCTGCTGCAAAGGCTGTTA-3’ |
|  | R: 5’-CTGGTTGTTGTCGTTACCGCT-3’ |
| Human-MCP | F: 5’-CATAGCAGCCACCTTCATTCC-3’ |
|  | R: 5’-TCTCCTTGGCCACAATGGTC-3’ |
| Human-IL-6 | F: 5’-GGCACCTCAGATTGTTGT-3’ |
|  | R: 5’-TAGTGTCCTAACGCTCATAC-3’ |
| Human-IL-1β | F: 5’-ATGATGGCTTATTACAGTGGCAA-3’ |
|  | R: 5’-GTCGGAGATTCGTAGCTGGA-3’ |
| Human-TNF-α | F: 5’-GAGGCCAAGCCCTGGTATG-3’ |
|  | R: 5’-CGGGCCGATTGATCTCAGC-3’ |
| Human-GAPDH | F: 5’-TGCACCACCAACTGCTTAGC-3’ |
|  | R: 5’-GGCATGGACTGTGGTCATGAG-3’ |
| Mouse-GAPDH | F: 5’-CTCATGACCACAGTCCATGC-3’ |
|  | R: 5’-CACATTGGGGGTAGGAACAC-3’ |
| hsa-miR-210-3p RT-primer | 5’-GTCGTATCCAGTGCAGGGTCCGAGGTATTCGCACTGGATACGA  CTCAGCC-3’ |
| hsa-miR-210-3p  (Stem-loop method) | F: 5’-CGCTGTGCGTGTGACAGC-3’ |
|  | R: 5’-AGTGCAGGGTCCGAGGTATT-3’ |
| hsa-miR-29a-3p RT-primer | 5’-GTCGTATCCAGTGCAGGGTCCGAGGTATTCGCACTGGATACGAC  TAACCG-3’ |
| hsa-miR-29a-3p  (Stem-loop method) | F: 5’-CGCGTAGCACCATCTGAAAT-3’ |
|  | R: 5’-AGTGCAGGGTCCGAGGTATT-3’ |
| U6 | F: 5’-CTCGCTTCGGCAGCACA-3’ |
|  | R: 5’-AACGCTTCACGAATTTGCGT-3’ |
| Cel-miR-39-3p  (Poly(A)-tailing method) | F: 5’-CACCGGGTGTAAATCAGCTTG-3’ |
| hsa-miR-210-3p  (Poly(A)-tailing method) | F: 5’-GCGTGTGACAGCGGCTGA-3’ |
| hsa-miR-29a-3p  (Poly(A)-tailing method) | F: 5’-CTAGCACCATCTGAAATCGGTTA-3’ |
| mmu-miR-210-3p  (Poly(A)-tailing method) | F: 5’-CTGTGCGTGTGACAGCGG-3’ |
| mmu-miR-29a-3p  (Poly(A)-tailing method) | F: 5’-ATTAGCACCATCTGAAATCGGTTA-3’ |

**Figure S1** Genotyping and experimental schedule for CREBZF floxed mice. **(A)** Genotyping of *CREBZF* floxed mice (318 bp allele; 248 bp wild-type), *ApoE* knockout mice (245 bp allele; 155 bp wild-type), and albumin-cyclization recombination (Alb-Cre) mice (400 bp) by PCR. **(B, C)** Diagram depicting the schedule of the *CREBZF*^fl/fl^ mouse experiments. This diagram was created by Figdraw.

**Figure S2** Enhanced expression of hepatic CREBZF in atherosclerotic mouse models. **(A)** Hepatic mRNA levels of CREBZF in wild-type (WT) and *ApoE*^-/-^ mice (8 weeks old, male, *n* = 4 mice per group) were quantified. **(B)** *ApoE*^-/-^ mice (8 weeks old, male, *n* = 3 mice per group) were fed either ND or HFD for 12 weeks, after which hepatic CREBZF mRNA expression was determined. ***P* < 0.01.

**Figure S3** Effect of hepatic CREBZF on cholesterol metabolism. **(A, B)** *CREBZF*^fl/fl^; *ApoE*^-/-^ and *CREBZF* LKO; *ApoE*^-/-^ mice (8 weeks old, male, *n* ≥ 5 mice per group) were fed ND or HFD for 12 weeks. (A) Measurement of the TC, TG, HDL, and LDL levels in the serum. (B) Measurement of the TC and TG levels in the liver. **(C)** *CREBZF*^fl/fl^; *ApoE*^-/-^ and *CREBZF* LKO; *ApoE*^-/-^ mice (8 weeks old, male, *n* ≥ 5 mice per group) were fed ND for 12 months. Measurement of the TC, TG, HDL, and LDL levels in the serum.

**Figure S4** Detection of hepatocytes with different CREBZF expression profiles. **(A)** Detection of CREBZF mRNA by q-PCR in HepG2 cells transfected with control or CREBZF adenovirus. **(B, C)** Detection of CREBZF protein by Western blot in HepG2 cells transfected with control or CREBZF adenovirus. **(D)** Detection of CREBZF mRNA by q-PCR in AML12 cells transfected with control or CREBZF-specific siRNA. **(E, F)** Detection of CREBZF protein by Western blot in AML12 cells transfected with control or CREBZF siRNA. ***P* < 0.01.

**Figure S5** Effects of hepatic CREBZF on the levels of inflammatory secretory proteins in the serum. **(A)** *CREBZF*^fl/fl^; *ApoE*^-/-^ and *CREBZF* LKO; *ApoE*^-/-^ mice (8 weeks old, male, *n* ≥ 5 mice per group) were fed ND or HFD for 12 weeks. ELISAs were used to analyze the levels of IL-6, TNF-α, MCP1, and IL-1β in the serum. **(B)** *CREBZF*^fl/fl^; *ApoE*^-/-^ and *CREBZF* LKO; *ApoE*^-/-^ mice (8 weeks old, male, *n* ≥ 5 mice per group) were fed ND for 12 months. ELISAs were used to analyze the levels of IL-6, TNF-α, MCP1, and IL-1β in the serum.

**Figure S6** Hepatocyte culture medium deprived of exosomes had no effect on endothelial inflammation. Culture medium from hepatocytes overexpressing CREBZF or control hepatocytes was collected, depleted of exosomes, and then applied to HUVECs. **(A)** q-PCR analysis of the gene expression of the inflammatory cytokines IL-6, TNF-α, and MCP1 in HUVECs. **(B)** Western blot analysis of ICAM1 and p-p65 protein expression in HUVECs.

**Figure S7** Effects of hepatocyte-derived exosomal miR-29a-3p on HUVECs. HUVECs were transfected with miR-29a-3p mimics or inhibitors following the manufacturer's protocol. **(A, B)** Following miR-29a-3p mimic treatment, the levels of miR-29a-3p, IL-6, MCP1, TNF-α, and IL-1β were determined by q-PCR. **(C, D)** The relative fold changes in ICAM1 and p-p65 protein expression following miR-29a-3p mimic treatment. **(E, F)** Following miR-29a-3p inhibitor treatment, the levels of miR-29a-3p, IL-6, MCP1, TNF-α, and IL-1β were determined by q-PCR. **(G, H)** The relative fold changes in ICAM1 and p-p65 protein expression following miR-29a-3p inhibitor treatment. ***P* < 0.01, ****P* < 0.001.
